# Ultrasensitive Detection of SARS-CoV-2 Spike Proteins Using the Thio-NAD Cycling Reaction: A Preliminary Study before Clinical Trials

**DOI:** 10.3390/microorganisms9112214

**Published:** 2021-10-25

**Authors:** Yuta Kyosei, Mayuri Namba, Daiki Makioka, Ayumi Kokubun, Satoshi Watabe, Teruki Yoshimura, Tadahiro Sasaki, Tatsuo Shioda, Etsuro Ito

**Affiliations:** 1 Department of Biology, Waseda University, Tokyo 162-8480, Japan; yuta.18-baseball@asagi.waseda.jp (Y.K.); myrnmb@asagi.waseda.jp (M.N.); mkokdik-3434@ruri.waseda.jp (D.M.); kok-ayumi@ruri.waseda.jp (A.K.); 2 Waseda Research Institute for Science and Engineering, Waseda University, Tokyo 169-8555, Japan; s.watabe@kurenai.waseda.jp; 3 School of Pharmaceutical Sciences, Health Sciences University of Hokkaido, Hokkaido 061-0293, Japan; yosimura@hoku-iryo-u.ac.jp; 4 Department of Viral Infections, Research Institute for Microbial Diseases, Osaka University, Osaka 565-0871, Japan; sasatada@biken.osaka-u.ac.jp (T.S.); shioda@biken.osaka-u.ac.jp (T.S.); 5 Graduate Institute of Medicine, School of Medicine, Kaohsiung Medical University, Kaohsiung 80756, Taiwan

**Keywords:** antigen test, COVID-19, SARS-CoV-2, spike protein, thio-NAD cycling, ultrasensitive ELISA

## Abstract

To help control the global pandemic of coronavirus disease 2019 (COVID-19), we developed a diagnostic method targeting the spike protein of the virus that causes the infection, severe acute respiratory syndrome coronavirus 2 (SARS-CoV-2). We applied an ultrasensitive method by combining a sandwich enzyme-linked immunosorbent assay (ELISA) and the thio-nicotinamide adenine dinucleotide (thio-NAD) cycling reaction to quantify spike S1 proteins. The limit of detection (LOD) was 2.62 × 10^−19^ moles/assay for recombinant S1 proteins and 2.6 × 10^6^ RNA copies/assay for ultraviolet B-inactivated viruses. We have already shown that the ultrasensitive ELISA for nucleocapsid proteins can detect ultraviolet B-inactivated viruses at the 10^4^ RNA copies/assay level, whereas the nucleocapsid proteins of SARS-CoV-2 are difficult to distinguish from those in conventional coronaviruses and SARS-CoV. Thus, an antigen test for only the nucleocapsid proteins is insufficient for virus specificity. Therefore, the use of a combination of tests against both spike and nucleocapsid proteins is recommended to increase both the detection sensitivity and testing accuracy of the COVID-19 antigen test. Taken together, our present study, in which we incorporate S1 detection by combining the ultrasensitive ELISA for nucleocapsid proteins, offers an ultrasensitive, antigen-specific test for COVID-19.

## 1. Introduction

Coronavirus disease 2019 (COVID-19) has significantly changed people’s lifestyles, economics, and medical service systems throughout the world. The widespread effects of the pandemic have provided opportunities for researchers to urgently develop useful diagnostics, effective vaccines, and specific drugs, not only for this pandemic, but future pandemics as well. In the present study, we aimed to improve antigen tests as an important diagnostic modality [1]. The severe acute respiratory syndrome coronavirus 2 (SARS-CoV-2) causing COVID-19 contains a single-stranded RNA (ssRNA) genome and four major proteins (spike proteins, nucleocapsid proteins, envelope proteins, and membrane proteins) [2]. The entry of SARS-CoV-2 into host cells is mediated by a transmembrane structural glycoprotein (i.e., spike protein) containing the N-terminal S1 fragment and the C-terminal S2 fragment [3,4]. The spike protein is a clove-shaped structure that protrudes from the surface of the virus, allowing the virus to bind to the host cell and undergo fusion. S1 is responsible for binding to the host cell receptor angiotensin-converting enzyme 2 (ACE2), whereas S2 promotes membrane fusion, which is responsible for the highly contagious property of SARS-CoV-2 [5].

The current gold standard for diagnosing COVID-19 is real-time polymerase chain reaction (PCR) [6]. However, there are many comments regarding the limitations of real-time PCR in the diagnosis, and these comments sometimes recommended combining chest computed tomography (CT) together with real-time PCR [7,8,9,10]. Chest CT has been established to play an important role in detecting lung abnormalities, allowing for precise treatment. Furthermore, real-time PCR may result in false negative results because viral RNAs are less stable than proteins [11,12] due to inadequate collection of clinical specimens or poor handling of a specimen during testing [12,13]. On the other hand, antigen tests are stable diagnostics for detection of past infection, infection progress, and transmission dynamics. Thus, stable, cost-effective, easy-to-use, and rapid diagnostic tests, such as antigen tests, are useful in addition to the diagnosis using real-time PCR [13]. In many antigen tests for COVID-19, the nucleocapsid proteins are utilized as the target antigen protein of SARS-CoV-2 [14,15]. The nucleocapsid proteins of SARS-CoV-2, however, are hardly distinguishable from those of conventional coronaviruses and SARS-CoV [16,17,18]. Thus, to avoid false-positive test results, a combination of tests against both the nucleocapsid and spike antigen proteins is strongly recommended [19], because the spike protein, which is the reason for the name coronavirus, is coronavirus-specific [20,21]. The binding affinity of the spike protein in SARS-CoV-2 with ACE2 is higher than that of SARS-CoV [22].

Detection methods specific for spike proteins recently emerged from several research groups, including ours [13,17]. Lee and colleagues developed a unique method for detecting SARS-CoV-2 spike S1 proteins using the SARS-CoV-2 ACE2 receptor, claiming that a suitable antibody pair for spike proteins was not available for a sandwich ELISA [13]. Their limit of detection (LOD) for S1 proteins was 0.05 ng/mL (ca. 10^−16^ moles/mL). On the other hand, our previous study using anti-S1 protein antibodies produced an assay with an LOD of ca. 10^−17^ moles/mL [17]. A highly sensitive method for detecting S1 proteins was achieved utilizing antibody tests, but the detection sensitivity could be further improved to achieve a more accurate diagnosis.

**Figure 1:**
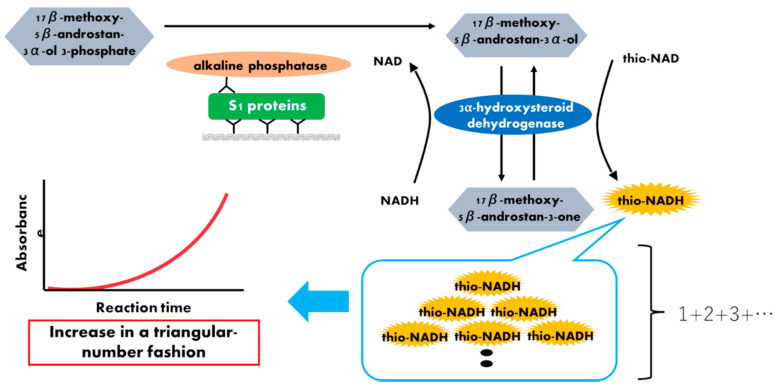
Schematic representation of a thio-NAD cycling ELISA for SARS-CoV-2 S1 proteins. Two antibodies used in ELISA specifically target the S1 protein. The first antibody is used to immobilize the protein, whereas the second antibody is labeled with alkaline phosphatase, which hydrolyzes a substrate containing phosphate. The hydrolyzed substrates are used in the thio-NAD cycling that employs a main enzyme (dehydrogenase) and its coenzymes (NADH and thio-NAD). Thio-NADH accumulates in a triangular manner and can be measured at 405 nm.

To produce an ultrasensitive method for detecting antigen proteins, we combined a sandwich enzyme-linked immunosorbent assay (ELISA) and thionicotinamide-adenine dinucleotide (thio-NAD) cycling [23,24]. In the thio-NAD cycling, a redox reaction is used as follows: a substrate, a derivative of androsterone 17β-methoxy-5β-androstan-3α-ol (i.e., 3α-hydroxysteroid), is oxidized to 17β-methoxy-5β-androstan-3α-one (i.e., 3-ketosteroid) via 3α-hydroxysteroid dehydrogenase (3α-HSD) using thio-NAD as a cofactor. In the reverse reaction, 17β-methoxy-5β-androstan-3α-one is reduced to 17β-methoxy-5β-androstan-3α-ol by 3α-HSD using NADH as the cofactor. Thio-NADH accumulates in a triangular-number manner during the cycling reaction (Figure 1). Here, we report a new protocol for an ultrasensitive thio-NAD cycling ELISA using new specific enzymes and antibodies for detecting SARS-CoV-2 S1. In addition, the viruses, which were prepared from ultraviolet B (UVB)-irradiated and inactivated SARS-CoV-2, were applied to this ultrasensitive thio-NAD cycling ELISA. Together with our previous ultrasensitive detection of nucleocapsid SARS-CoV-2 proteins [25], an accurate, ultrasensitive antigen test for COVID-19 was developed.

## 2. Materials and Methods

### 2.1. Materials, Reagents, and Chemicals

An ultrasensitive thio-NAD cycling ELISA uses 2 types of antibodies (i.e., capture and detection). The capture-side antibody and the detection-side antibody for the SARS-CoV-2 spike protein were provided by Hakarel (Clone #A1-1C7 and #A2-7G6, respectively). The recombinant antigen for SARS-CoV-2 spike S1 protein was obtained from GenScript (human cell source, 78.3 kDa, Cat #Z03501). Alkaline phosphatase (ALP) and nicotinamide adenine dinucleotide (NADH) were purchased from Roche. The thio-NAD was obtained from Oriental East. 3α-hydroxysteroid dehydrogenase (3α-HSD) was purchased from Asahi Kasei Pharma. 17β-methoxy-5β-androstan-3α-ol 3-phosphate was synthesized by one of the authors (T.Y.). SARS-CoV-2 (JPN/TY/WK-521) was propagated in Vero-E6/TMPRSS2 (JCRB1819) cells using Dulbecco’s modified Eagle’s medium (DMEM) solution with 2% fetal calf serum, with a viral infectivity of 5.25 log_10_ TCID_50_/50 μL and an RNA amount of 2.6 × 10^7^ RNA copies/μL. The culture supernatant (hereafter referred to as viruses) was inactivated by UVB irradiation at 51 μW/cm^2^ for 30 min (GL15; Toshiba Lighting & Technology). The other chemicals and disposable plasticware were of commercial grade.

### 2.2. Ultrasensitive Thio-NAD Cycling ELISA

Although similar experiments for the SARS-CoV-2 recombinant S1 protein were achieved using an ultrasensitive thio-NAD cycling ELISA [17], in the present study we attempted to improve the detection sensitivity of the recombinant protein by applying our method to viruses. For this purpose, we used another 3α-HSD from Asahi Kasei Pharma [26] and a new combination of antibodies for the anti-S1 protein from Hakarel, as described in Section 2.1. Our ultrasensitive thio-NAD cycling ELISA was based on a sandwich ELISA and developed by Watabe and Ito [23,27]. In the first phase, a 100 μL solution of primary antibody, adjusted to 1 μg/mL in 50 mM Na_2_CO_3_ (pH 9.6), was added to microplate wells and incubated at room temperature for 1 h. The microplates were then incubated with 1% bovine serum albumin (BSA) in Tris-buffered saline (TBS) at room temperature for 1 h. S1 protein (100 μL, i.e., antigen) was added to each well and incubated at room temperature for 1 h with shaking. In the second phase, the antigen samples were diluted with TBS containing 0.1% BSA. A 100 μL solution of secondary antibody conjugated with ALP and adjusted to 100 ng/mL in TBS including 0.05% Tween 20 and 0.1% BSA was then added to the wells and incubated at room temperature for 1 h with shaking. To amplify the ELISA signal, 100 μL of thio-NAD cycling solution was added to each well. This thio-NAD cycling solution contained 1.0 mM NADH, 2.0 mM thio-NAD, 0.4 mM 17β-methoxy-5β-androstan-3α-ol 3-phosphate, and 10 U/mL 3α-HSD in 100 mM Tris-HCl (pH 9.0). In thio-NAD cycling, thio-NADH was measured with a microplate reader (Corona Electric SH-1000) at 405 nm (Figure 1). The 405 nm signals were normalized to those at 660 nm.

### 2.3. Statistical Analysis

The experimental data were obtained by subtracting the mean value of the blank signals from each of the corresponding measured datapoints. The LOD was estimated from the mean of the blanks, the standard deviation (SD) of the blanks, and a confidence factor of 3. The limit of quantitation (LOQ) was estimated by the same method used to estimate the LOD, but with a confidence factor of 10 [28]. The coefficients of variation (CVs) for spike protein antigen were obtained in the assessments of intra-assay and inter-assay reproducibility. The data are expressed as mean ± SD. Significant differences were determined using FreeJSTAT Version 22.0E (http://toukeijstat.web.fc2.com/EnglishPage.html (accessed on 9 September 2021), with *p* < 0.05 considered significant.

## 3. Results

### 3.1. Measurement of Recombinant S1 Protein

**Figure 2:**
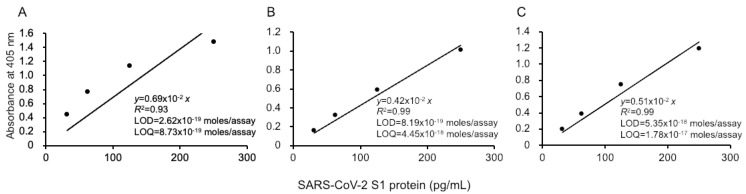
Linear calibration curves of SARS-CoV-2 S1 proteins by thio-NAD cycling ELISA. Three datasets measured by 3 investigators are presented as (**A**–**C**). The absorbance was obtained from a 40 min cycling reaction time. Each investigator performed 3 replicate experiments. The dots show the measured plots, and the lines show those obtained by the least-squares method. The antigen was applied in the range of 31.25–1000 pg/mL.

Our ultrasensitive thio-NAD cycling ELISA was applied to evaluate the LOD and LOQ for the SARS-CoV-2 recombinant S1 protein (Figure 2). Three investigators obtained linear calibration curves of the recombinant S1 proteins ranging from 31.25 to 1000 pg/mL. The absorbance of thio-NADH was measured after 40 min of thio-NAD cycling in all cases by the three investigators. One linear calibration curve was expressed as *y* = 0.0069*x*, *R*^2^ = 0.93 (Figure 2A). The LOD obtained from this calibration curve was 2.62 × 10^−19^ moles/assay, and the minimum LOQ was 8.73 × 10^−19^ moles/assay (*n* = 3). Because the molecular mass of the antigen (i.e., recombinant S1 protein) was 78.3 kDa and the assay volume was 100 μL, the LOD and LOQ corresponded to 0.205 and 0.683 pg/mL, respectively. The intra-assay CV was 1.4% for 1000 pg/mL (*n* = 3).

The second and third investigators obtained the following linear calibration curves. That obtained by the second experimenter was expressed as *y* = 0.0042*x*, *R*^2^ = 0.99 (Figure 2B). The LOD was 8.19 × 10^−19^ moles/assay, and the minimum LOQ was 4.45 × 10^−18^ moles/assay (*n* = 3). The intra-assay CV was 0.8% for 1000 pg/mL (*n* = 3). The linear calibration curve obtained by the third investigator was expressed as *y* = 0.0051*x*, *R*^2^ = 0.99 (Figure 2C). The LOD was 5.35 × 10^−18^ moles/assay, and the minimum LOQ was 1.78 × 10^−17^ moles/assay (*n* = 3). The intra-assay CV was 0.7% for 1000 pg/mL (*n* = 3). In addition, the data obtained by the three investigators showed that the CV in the inter-assay reproducibility was 15% by 1000 pg/mL (*n* = 3).

### 3.2. Measurement of SARA-CoV-2 Viruses

**Figure 3:**
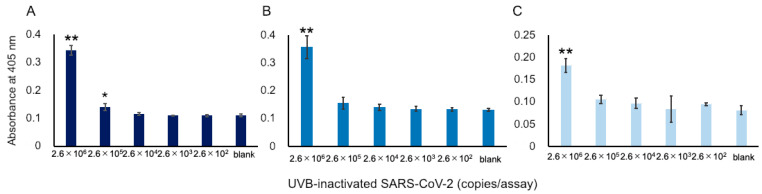
Detection of S1 proteins in UVB-inactivated SARS-CoV-2. Because a SARS-CoV-2 virus contains single-stranded RNA, the RNA copies in the *x*-axis correspond to the number of viruses. Three datasets measured by 3 investigators are presented as (**A**–**C**). The absorbance was obtained from a 60 min cycling reaction time. Each investigator performed 3 replicate experiments. Error bars indicate the standard deviation. The 3 investigators consistently obtained signals that were higher than the blank at concentrations over 2.6 × 10^6^ RNA copies/assay. * *p* < 0.05, ** *p* < 0.01 compared with the value of blank by one-way ANOVA with a post-hoc Tukey test.

We attempted to apply our ultrasensitive thio-NAD cycling ELISA to detect SARS-CoV-2 viruses. As described in Section 2.1, we prepared UVB-irradiated, inactivated viruses to perform the experiments without requiring a biosafety level 3 facility. This inactivation procedure was necessary for us to be able to conduct experiments in our laboratory. In the past, when we used the previous commercially available antibodies, we failed to detect UVB-inactivated viruses [17]. Using the Hakarel antibodies, three investigators independently performed the experiments using UVB-inactivated viruses. For the experiments, the viruses were serially diluted from 2.6 × 10^7^ RNA copies/μL. The absorbance of thio-NADH was measured after 60 min of thio-NAD cycling. Only at concentrations greater than 2.6 × 10^6^ RNA copies/assay did all three investigators consistently obtain signals higher than those of the blank (*n* = 3 each, *p* < 0.05 by one-way ANOVA with a post-hoc Tukey test; Figure 3).

## 4. Discussion

The present study could completely improve the data obtained by the previous one. The most important improvement is that the new antibodies used in the present study can recognize the UVB-inactivated SARS-CoV-2, whereas the antibodies used in the previous study [17] cannot recognize these viruses. Regarding the recombinant S1 protein, our previous study using another set of anti-S1 protein antibodies and another 3α-HSD produced an LOD of 2.4 × 10^−18^ moles/assay and an LOQ of 7.5 × 10^−18^ moles/assay [17]. Our present assay to quantify a recombinant S1 protein (i.e., LOD: 2.62 × 10^−19^ moles/assay; LOQ: 8.73 × 10^−19^ moles/assay) was 1 order of magnitude more sensitive than the previous assay. To our knowledge, this detection limit for S1 proteins of SARS-CoV-2 has not been achieved by any other research groups.

In ultrasensitive measurements, an improvement of 1 order of magnitude is very important [29]. This difference was given by the data obtained from three replicate experiments by three different investigators. These three investigators were well trained in the ELISA experiments. The result of “1 order of magnitude more sensitive” is critical. This result is thought to be due to the higher specificity of the antibodies and the greater stability of 3α-HSD used in our present study. Furthermore, we need to add a comment on the word “ultrasensitive”, which sometimes causes confusions in science [29]. Gooding defined an ultrasensitive bioanalytical sensor as “a biosensor with sufficient sensitivity and low background to allow subpicomolar detection limits” [29]. Thus, the LOD with 10^−13^ moles/liter or smaller can be referred to as an “ultrasensitive” detection. When we use a 96-well microplate for ELISA, one assay volume may be 100 μL. Therefore, the value of 10^−17^ moles/assay might be a challenging detection sensitivity. Our own ELISA attempts to detect proteins at zeptomolar detection limits, i.e., 10^−21^ moles/assay [26]. We believe that our system can be called an “ultrasensitive” ELISA.

On the other hand, regarding the minimum detection limit for viruses, the value of 2.6 × 10^6^ RNA copies/assay was not sufficiently sensitive, which may be due to the following two reasons: (1) When we used another set of antibodies against the S1 protein in our previous study [17], we failed to detect UVB-irradiated and inactivated SARS-CoV-2. Thus, the antibodies used in the present study are more suitable for antigen tests. (2) In our other previous experiments to target the SARS-CoV-2 nucleocapsid protein, the results obtained from our ultrasensitive thio-NAD cycling showed a minimum detection limit for viruses of 2.6 × 10^4^ RNA copies/assay [25]. In other words, simultaneous detection of spike and nucleocapsid protein detection enabled us to detect SARS-CoV-2 at the level of 10^4^ viruses/assay. This simultaneous detection offers not only good detection sensitivity, but also avoids false positive testing results, as suggested by Werner et al. [19].

In any case, we achieved a 2-order of magnitude difference between the assay for the nucleocapsid protein (i.e., 10^4^ viruses/assay) and that of the spike protein (i.e., 10^6^ viruses/assay). We considered this difference to be due to the power and duration of the UVB irradiation, because UVB irradiation damages the spike proteins to a much greater extent than the nucleocapsid proteins. Very recently, Loveday and colleagues evaluated the conditions for inactivating SARS-CoV-2 using UV irradiation (UVC rather than UVB) and heat treatment [30]. Their purpose was the same as ours because they also wanted to avoid using biosafety level 3 facilities to study SARS-CoV-2. They reported that the detection sensitivity of their ELISA for the spike proteins changed according to the UV irradiation duration. This result suggests that the anti-S1 antibodies in our previous study [17] would be capable of recognizing the UVB-inactivated viruses, if the UVB irradiation duration was shortened. Therefore, if the UVB irradiation technique is also used in the future to inactivate the viruses (i.e., clinical samples), further studies are needed to determine the optimal conditions for the power and duration of UVB. To us, however, the aim of our present study shows the fact that the spike proteins can be detected using our technique, and we want to insist that it is very rare to detect them.

When we start the preparation of the primary (i.e., capture) antibody on microplates and if we need to obtain the champion data, the turn-around time of our assay is 4 h or so. However, when we apply our assay to the real, clinical patients’ samples, we can offer the microplates prepared with primary antibody to doctors or patients, and thus we can skip this procedure time. Furthermore, the time for thio-NAD cycling is determined as a “trade-off” way between its time and the detection sensitivity—that is, if it is enough to detect one-order worse sensitivity than the present study, the detection time is only 10 min [17]. The further reduction in the total time, including the incubation time with samples and secondary (i.e., detection) antibody, may be achieved, if our assay will be distributed as a commercially available kit.

We have to note that the antibodies used in the present study can cross-react to the variants of concern of SARS-CoV-2. Recently, the Delta variant of concern has emerged as the dominant strain internationally [31,32]. It was reported to be more transmissible than other variants [33], and the vaccines might be less effective in preventing infection than they are with the Alpha variant of concern [34,35]. The antibody, Clone #A1-1C7, used in the present study was confirmed to react to the Delta variant of concern. This result will be reported elsewhere by the developer, Hikaru Sonoda of Hakarel.

We should comment on the other methods to detect proteins in SARS-CoV-2. The nucleocapsid proteins in SARS-CoV-2 detected by liquid chromatography/tandem mass spectrometry (LC-MS/MS) were usually at the levels of 10^−16^ moles/μL, and thus Kipping et al. has recently improved the detection sensitivity to the levels of 10^−18^ moles/μL in their LC-MS/MS study [36]. If it is allowed to change “per μL” to “per 100 μL” (i.e., our one assay volume), the detectable molecule number is 10^−16^ moles/100 μL. In addition, the pandemic of COVID-19 has also set the trend of developments of various point-of-care (POC) diagnostics using new techniques [37]. These techniques include methods such as surface-enhanced Raman scattering (SERS)-based methods, surface plasmon resonance (SPR)-based methods, lab-on-a-chip (LOC) methods, lab-on-a-disc (LOAD) methods, microfluidic paper-based analytical devices (μPADs), miniaturized PCR methods, and isothermal nucleic acid amplification (INAA) methods. We expect that these POC diagnostics show the advantages to the speed, sensitivity and specificity, cost, and ability of on-site detection.

## 5. Conclusions

It is difficult to detect the spike proteins of SARS-CoV-2 [13], but the simultaneous detection of nucleocapsid proteins and spike proteins is suitable for COVID-19 antigen tests [19]. As a follow-up to our previous study of the detection of SARS-CoV-2 nucleocapsid proteins, our present study developed an ultrasensitive thio-NAD cycling ELISA to detect SARS-CoV-2 spike proteins, which were prepared from UVB-irradiated and inactivated viruses. To achieve this, we used a different 3α-HSD for the thio-NAD cycling and a new set of anti-S1 antibodies. One limitation of our study is that the UVB irradiation virus inactivation protocol requires further optimization to better detect the spike proteins, which will be achieved in future work. More recently, an ELISA with europium enhancement has been proposed to detect the recombinant spike and nucleocapsid proteins and the SARS-CoV-2 viruses [38]. Even though the determination method of LOD is different from ours, our LOD is considerably better.

## Data Availability

Data are contained within the article.
